# Evolution of poled state in P(VDF-TrFE)/(Pb,Ba)(Zr,Ti)O_3_ composites probed by temperature dependent Piezoresponse and Kelvin Probe Force Microscopy

**DOI:** 10.1038/s41598-017-18838-1

**Published:** 2018-01-10

**Authors:** V. V. Shvartsman, D. A. Kiselev, A. V. Solnyshkin, D. C. Lupascu, M. V. Silibin

**Affiliations:** 1 Institute for Materials Science and Center for Nanointegration Duisburg-Essen (CENIDE), University of Duisburg-Essen, Universitätsstraße 15, 45141 Essen, Germany; 2 National University of Science and Technology “MISiS”, 119049 Moscow Leninskiy pr. 4, Russia; 3 Department of Condensed Matter Physics, Tver State University, 170100 Tver, Russia; 4 National Research University of Electronic Technology “MIET”, Bld. 1, Shokin Square, 124498 Moscow, Russia

## Abstract

Polarized states of polymer/inorganic inclusion P(VDF-TrFE)-(Pb,Ba)(Zr,Ti)O_3_ composites are studied at the nanoscale using both piezoresponse force microscopy (PFM) and Kelvin probe force microscopy (KPFM). It has been shown that inorganic inclusions can be visualized using KPFM due to a discontinuity of the surface potential and polarization at the interface between the inclusions and the polymer matrix. The temperature evolution of the PFM and KPFM signal profiles is investigated. Softening of the polymer matrix on approaching the Curie temperature limits application of the contact PFM method. However non-contact KPFM can be used to probe evolution of the polarization at the phase transition. Mechanisms of the KPFM contrast formation are discussed.

## Introduction

During the last years, there has been a growing interest to develop polar materials having low weight and good mechanical flexibility together with significant piezoelectric, dielectric, and pyroelectric responses^1–6^. Such materials find applications in modern electronics devices such as acoustic transducers, microelectromechanical systems (MEMS), memory elements, infrared sensors, thermal imagers, hybrid electric vehicles, and energy storage devices. Among them are composites consisting of polymer matrix and ferroelectric ceramic grains. They maintain mechanical elasticity, low acoustic impedance and high dielectric strength of the polymers, but attain large pyro- and piezoelectric coefficients of ferroelectric ceramics. Of particular interest are composites having a polar polymer matrix based on poly(vinylidene fluoride), PVDF, and its copolymers^1,4,6–8^. Due to the relatively large dielectric permittivity of polar polymers^9^, the embedded ferroelectric ceramic grains can be easily polarized providing high degrees of poling and large electromechanical and pyroelectric activity. Moreover, it is possible to polarize matrix and fillers in the same, as well as in opposite directions, which opens a new degree of freedom to vary the properties of the composites. Polymer composites filled with ceramic particles featuring high-dielectric-constants, low dielectric loss, and good compatibility with printed-circuit-boards are promising candidates for embedded capacitors used in embedded passive technology^10^. Incorporation of ceramic fillers into the polymer matrix results in composites of large dielectric permittivity and high electric breakdown strength. This makes such composites favorable for applications demanding high electric energy density such as energy storage devices in pulse power technology, mobile electronic devices, hybrid electric vehicles, etc.^11,12^.

The most attractive ferroelectric polymer materials are copolymers of vinylidene fluoride and trifluoroethylene, P(VDF-TrFE). Contrary to the pure polyvinylidene fluoride, PVDF, these materials have a high degree of crystallinity, i.e. ratio between ordered and randomly arranged molecules in the polymer. They show ferroelectricity already in the as-grown state, which causes their good piezoelectric and pyroelectric properties^9^. Improvement of the piezoelectric, dielectric and elastic properties of P(VDF-TrFE) polymers has been achieved by mixing them with electroactive fillers, like triglycine sulphate crystals^8^, lead zirconate titanate^1,7,13^, barium titanate^14^ ceramics, as well as some relaxor ferroelectrics^15,16^.

Local heterogeneities of functional properties, which are characteristic for composite materials, can significantly affect the macroscopic performance. Therefore, probing of such heterogeneities is highly demanded. Such studies can be done using scanning probe microscopy (SPM) based methods probing electrical and mechanical properties of materials at the nanometer scale i.e. with resolution comparable to the size of the interfaces in the composites^17,18^. There have been only a few reports on SPM based studies of ferroelectric polymer/ceramic composites^19–22^. Using piezoresponse force microscopy (PFM), local polarization reversal was studied in BaTiO_3_/PVDF composite nanofibers^20^ and (Na,K)NbO_3_ nanoparticle-embedded P(VDF-TrFE) nanofiber composites^21^. Silibin *et al*. showed that piezoresponse of the polymer matrix is enhanced in vicinity of lead zirconate titanate inclusions due to inhomogeneous stress field originating form a strong difference between the thermal expansion coefficients of the composite constituents^22^.

In this paper we report on microscopic spatially resolved studies of poled states in a P(VDF-TrFE)-(Pb,Ba)(Zr,Ti)O_3_ composite film with 40 vol.% of (Pb,Ba)(Zr,Ti)O_3_. Temperature evolution of artificially created domains was investigated using both piezoresponse force microscopy (PFM) and Kelvin probe force microscopy (KPFM).

## Results

**Figure 1:**
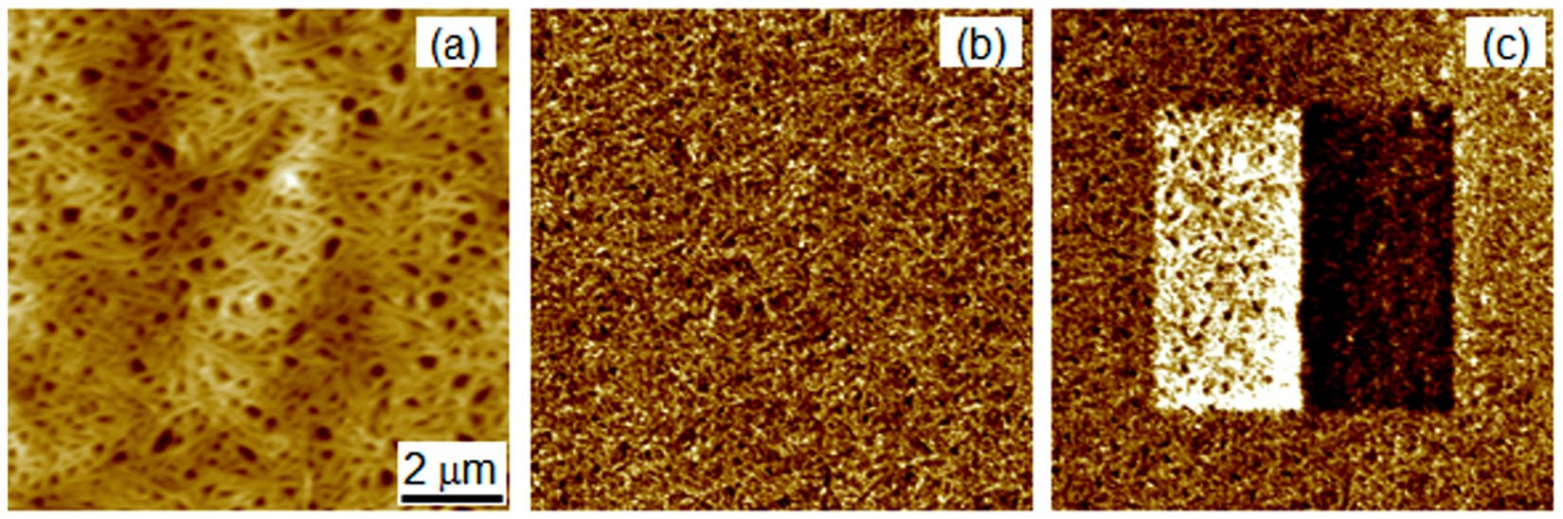
Topography (**a**), PFM images of a pristine state (**b**) and after poling by +/−60V (**c**).

Figure 1 shows the topography and PFM images of a composite PVDF-TrFE/BPZT 60/40 film in the as-prepared state and after poling with ± 60 V. The topography images shows that the polymer matrix keeps its chain structure in spite of relatively high amount of inorganic filler^23^.

The poling was performed by scanning of a selected area under a bias voltage applied to the tip. One can clearly see that two domains with oppositely oriented polarization were created. Here the dark and bright contrast corresponds to the polarization oriented upward and downward relative to the sample surface, respectively. The created domains were stable: no decrease of the piezoresponse contrast and decay of the poled area was observed at least for 24 hours after poling.

**Figure 2:**
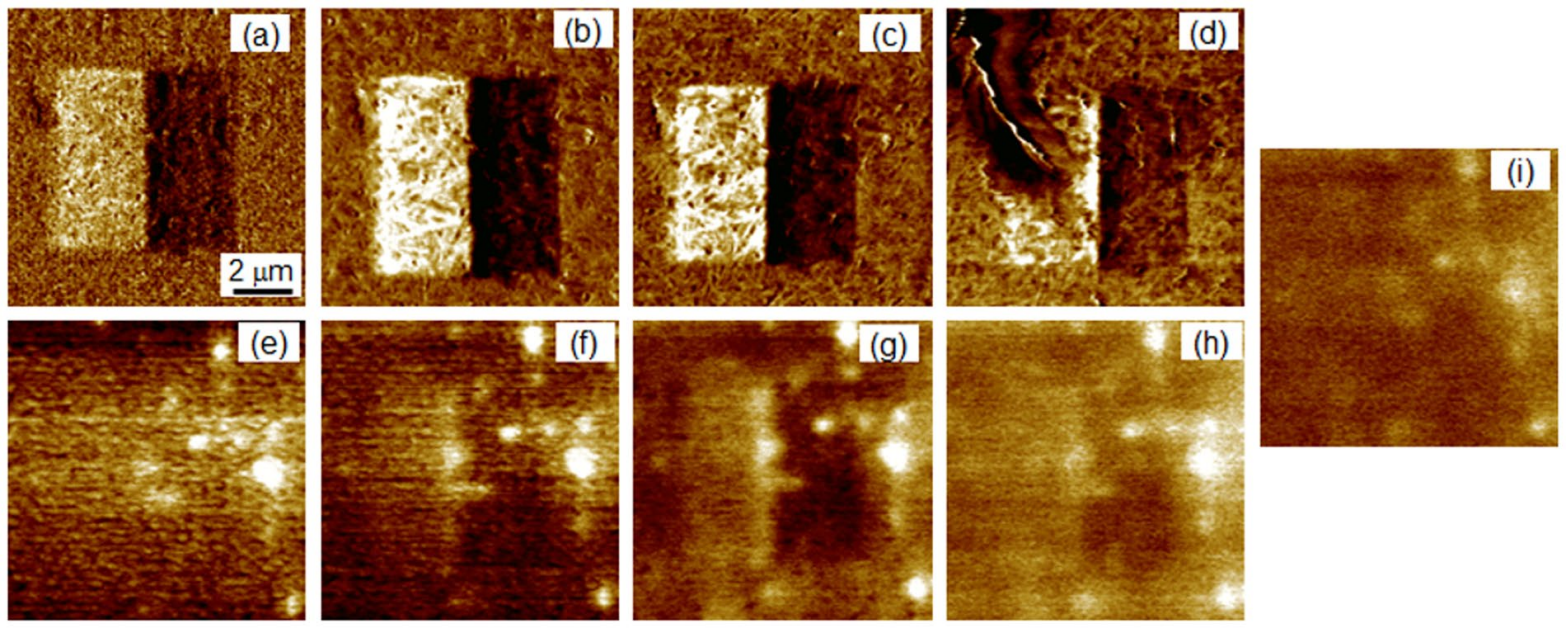
PFM (**a–d**) and KPFM (**e–i**) images of a poled area taken at 301 K (**a,e**), 333 K (**b,f**), 353 K (**c,g**), 373 K (**d,h**), and 393 K (**i**).

Figure 2(a–d) presents the PFM images of the poled region recorded up on heating between room temperature and 373 K. Unfortunately above approximately 363 K strong changes of the topography of the scanned regions were observed, which were attributed to approaching the melting temperature and correspondingly a strong softening of mechanical properties of the film. Namely, the amorphous fraction of the polymer matrix is softened, while the crystalline fraction responsible for the ferroelectric properties remains stable up to the melting temperature. Since PFM is a contact method, the tip started to damage the surface. Obviously this limits applicability of contact methods to investigate the phase transition range in the studied materials.

To overcome this problem we applied the non-contact KPFM method. Figure 2e shows the KPFM images of the same poled region, where the PFM images were taken. By comparison between the PFM and KPFM images one can see that the KPFM image taken at room temperature did not show a contrast between the positively and negatively poled regions. At the same time, spots showing a brighter contrast, i.e. a larger surface potential relative to their environment were seen. Such contrast inhomogeneities are not visible in the PFM images. We attribute them to BPZT grains, which exhibit a jump of the electrostatic potential at the interface to the polymer matrix. The size of the BPZT grains estimated from the KPFM images is about 1 µm and agrees well with data of scanning electron microscopy (Suppl. Figure 1). It seems that these grains are located at a certain depth from the film surface, therefore they can not be probed by PFM. Indeed, the dielectric permittivity of P(VDF-TrFE) (*ε* ≈ 10) is much smaller than the dielectric permittivity of BPZT (*ε* ≈ 2000). As a result, the applied electric field drops very fast in the low-permittivity matrix and does not penetrate much into a buried PBZT grain. Therefore we can not induce a substantial electromechanical deformation to be detected by PFM. Otherwise, due to different values of the remanent polarization in PVDF (*P*_*r*_ ≈ 40 mC/m^2^) and BPZT (*P*_*r*_ ≈ 100 mC/m^2^) there is a jump of the polarization at the interface between two phases and related bound charges should result in a jump of surface potential. In such a way the KPFM method seems sensitive enough to probe the distribution of buried particles in our composite. The question how deep particles can still be resolved remains open.

While the KPFM contrast between two oppositely polarized region was not seen at room temperature, it was revealed on heating above 333 K and then disappeared again above 393 K, which approximately corresponds to the Curie temperature of the copolymer matrix^24,25^. (Fig. 2f–i). The later observation confirms that the contrast observed by KPFM is related to the ferroelectric polarization. To investigate the evolution of the KFPM contrast in more details we studied the temperature dependences of several KPFM signal profiles taken in various regions.

**Figure 3:**
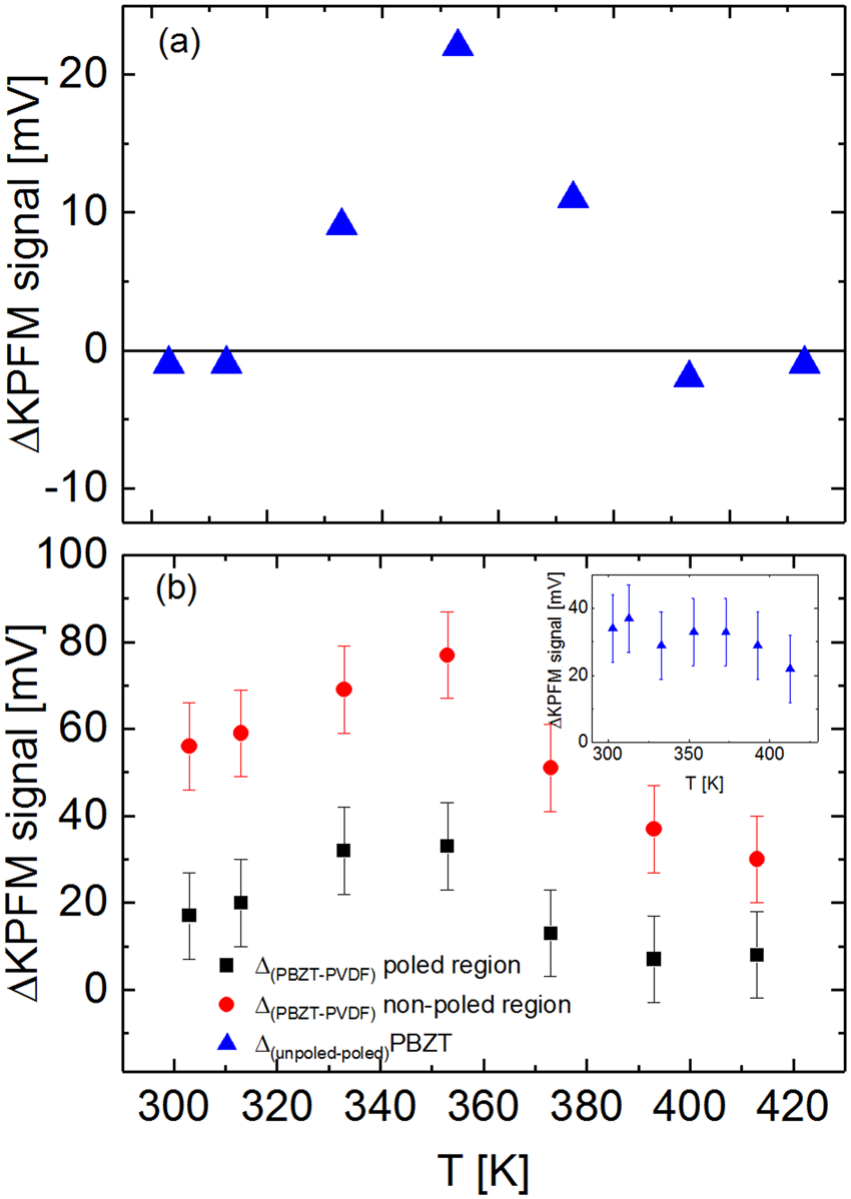
(**a**) Difference between the KPFM signal in the in the positively poled and negatively poled areas. (**b**) Difference between the KPFM signal for PBZT particles and the P(VDF-TrFE) matrix in the poled area (squares); in the non-poled area (circles); an insert shows the difference between the KPFM signals of PBZT particles in the poled and non-poled areas (triangles).

Figure 3a shows a difference between KPFM signals taken in the regions poled by negative and positive biases. One can see that the effect of poling is minor at room temperature. Then on heating above 333 K the difference between the KPFM signals in positively and negatively poled areas appeared and increased reaching the maximum value at 353 K. At higher temperature the difference started to decrease and disappeared at approximately 393 K. Figure 3b shows the difference between the KPFM signals of BPZT inclusions and the polymer matrix. For inclusions both inside and outside the poled region ΔKPFM first slightly increased, reaching a maximum at 333–353 K and decreasing at higher temperatures. The difference between the KPFM signal of the BPZT inclusions inside and outside the poled area is practically temperature independent.

## Discussion

**Figure 4:**
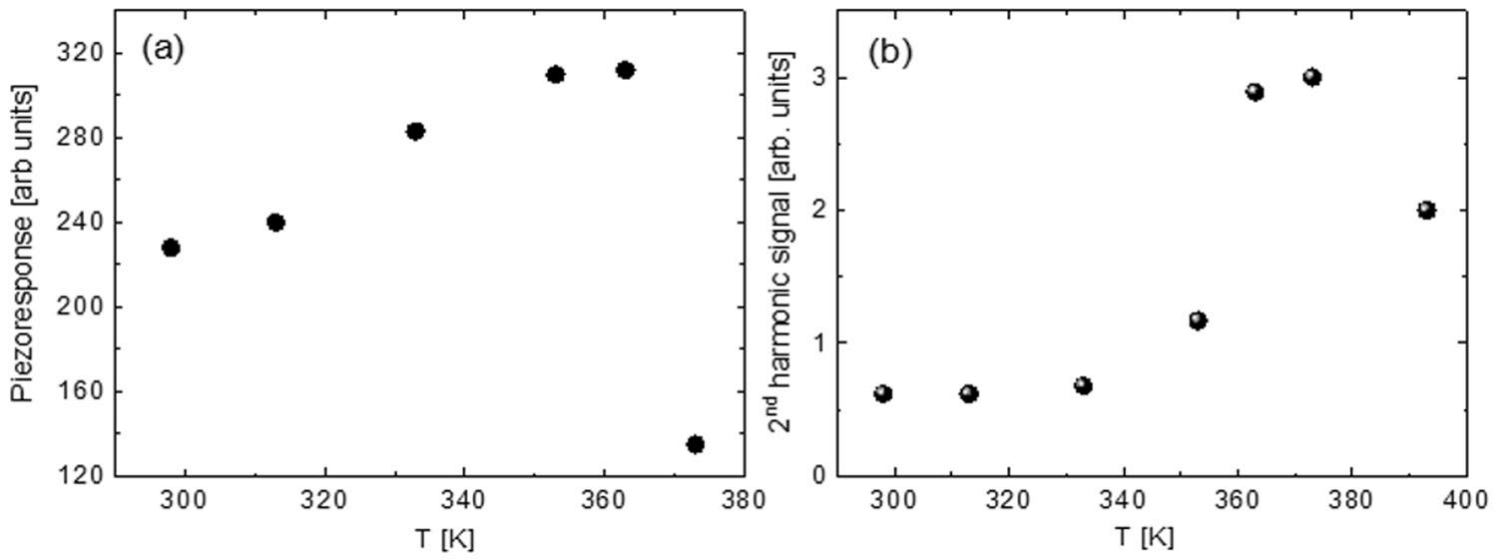
Temperature dependences of the 1^st^ harmonic (piezoresponse) (**a**) and the 2^nd^ harmonic (**b**) of the PFM signal measured inside the poled area.

From analysis of PFM images we can conclude that a) scanning under *dc*-bias results in formation of a homogeneously poled area and b) piezoresponse of the polymer matrix is barely affected by the presence of the BPZT grains. To evaluate the variation of the piezoresponse with temperature we compare the average intensity of the PFM signal in negatively and positively poled regions. To this extent we averaged over 50 cross-section lines drawn across these regions. As one can see in Fig. 4a the piezoresponse is growing upon heating and reaches its maximum at approximately 363 K. The strong drop of the piezoresponse at higher temperature can be due to both transition into the paraelectric phase and degradation of scanning condition on approaching the melting temperature of the polymer matrix. Figure 4b shows the temperature dependences of the 2^nd^ harmonic PFM signal measured in the same region. There are two contributions to this signal^26^. First, the electrostatic contribution that is proportional to the local dielectric permittivity. Second, the electrostriction contribution that is proportional to the square of the dielectric permittivity. Therefore one can expect that the variation of the 2^nd^ harmonic PFM signal reflects the temperature dependence of the local dielectric permittivity. Indeed, we observed a sharp maximum of the 2^nd^ harmonic PFM signal close to the Curie temperature of the polymer matrix and its temperature dependence is qualitatively similar to the temperature dependence of the high-frequency dielectric permittivity for this compound^27^.

The observed variation of the KPFM contrast between the polarized regions can be rationalized as follows. Scanning using a *dc* biased tip results not only in polarization reversal but also in injection of free charge carriers that tend to screen the bound charges related to the polarization. Absence of the KPFM contrast between the “up” and “down” polarized domains indicates that contributions from the screening charges and bound charges, which have the opposite signs, compensate each other. On heating the up and down polarized domains start to show more positive and more negative KPFM signal, respectively, which indicates an excess of the positive charge for the up polarized domain and excess of the negative charge for the down polarized domain. That means that amount of the screening free charges is decreased and the KPFM contrast is dominated by the bound charges. The decay of screening charges is a thermally activated process. Above a certain temperature the KPFM contrast starts to fade out again, which is due to the decay of polarization on approaching the Curie temperature (*T*_*C*_ ~ 393 K).

The distribution of the KPFM signal between the BPZT particles and the polymer matrix can be explained in the following way. There is a difference between the work functions of the P(VDF-TrFE) matrix and a BPZT inclusion resulting in the formation of a space charge at the interfaces^28^, which provides a contrast in the KPFM image. Of cause, intensity of the contrast depends on the depth position of the particle. A discontinuity of polarization between the top PVDF layer and a submerged BPZT grain may also result in a local excess of bound charges. Indeed, we observed that the KPFM contrast between the BPZT grains and the polymer matrix decays on approaching the Curie temperature of the polymer matrix. Interestingly, in the poled region the contrast initially increases on heating that might be related to thermal activation and release of screening charges that were injected from the tip during the poling procedure. Simultaneously, the difference between the surface potential of grains inside and outside the poled areas is practically temperature independent. The BPZT grains in negatively and positively poled areas show similar KPFM signal. This may indicate that the polarization of BPZT grains remains unaltered by poling. Indeed, an applied *dc* bias mainly drops in the top polymer layer and might not be enough to polarize the embedded ceramic grains.

## Conclusions

Local piezoelectric and electronic properties of the P(VDF-TrFE)/BPZT 60/40 composite were studied using piezoresponse force microscopy and Kelvin probe force microscopy. We found that the subsurface BPZT particles can be visualized using KPFM due to a mismatch of the work function and the polarization discontinuity at the polymer-particle interfaces. Moreover KPFM can be used for investigation of the temperature evolution of the poled state across the phase transition. Due to approaching the melting temperature and softening of the polymer matrix, the contact PFM method can only be applied in a limited way.

## Methods

A composite film containing 60 volume % of the random copolymer vinylidene fluoride with trifluoroethylene P(VDF-TrFE) as a matrix and 40 volume % of barium lead zirconate titanate Pb_0.75_Ba_0.25_(Zr_0.53_Ti_0.47_)O_3_ (BPZT) ceramic inclusions was studied. Incorporation of Ba into PZT^29^ improves electromechanical and dielectric properties of PZT^30,31^. Thus, we expect a stronger impact on properties of the composites as compared to using pure PZT grains. For sample preparation, Solef® P(VDF-TrFE) copolymer powder grade 2P001 (Solvay S.A.) with an TrFE content of about 30% was used. The powder was dissolved in a dimethylsulfoxide (DMSO)/acetone solution. The commercial BPZT ceramic powder with a grain size of ~ 1 μm (VA-650, “Avrora-Elma”, Volgograd, Russia) was added to the solution containing the copolymer. The films were cast from the solutions to obtain samples with a thickness of ~ 50–60 μm. After film formation, the samples were not preliminarily polarized. More detailed information about samples preparation can be found elsewhere^22,32^.

Scanning electron microscopy shows that BPZT particles with size of about 1 µm are uniformly distributed across the film thickness (Suppl. Figure 1). Some agglomeration of the BPZT particles takes place.Dielectric permittivity measurements confirm that the polymer matrix in the composite film keeps its ferroelectric state. The ferroelectric-paraelectric phase transition in the composite film occurs approximately at the same temperature as in the pure polymer film (Suppl. Figure 2).

Scanning probe experiments were carried out with a commercial atomic force microscope MFP-3D (Asylum Research, USA). Two different methods were used: PFM and KPFM. The PFM method is based on the detection of local deformations of a piezoelectric material induced by a weak *ac* voltage applied to the conductive tip being in contact with the sample surface. The amplitude of the 1^st^ harmonic of these deformations is proportional to the local piezoelectric coefficients, while its phase depends on the orientation of the polarization vector relative to the normal to the surface. Thus, PFM allows to distinguish ferroelectric domains with oppositely oriented polarization as regions with different contrasts in the piezoresponse image. The PFM measurements were performed under an applied *ac* voltage with the amplitude *V*_ac_ = 5 V and frequency *f* = 50–150 kHz. The KPFM method is sensitive to the local surface potential. For ferroelectrics the surface potential depends on the sign of the bound polarization charges^33^. The screening charges also affect the potential distribution^33^. Both PFM and KPFM measurements were performed using NSG10 cantilevers with the PtIr coated conducting tip: tip apex radius *R* ~ 20 nm, cantilever stiffness *k* ~ 12 N/m, and resonance frequency *f* ~ 240 kHz. The measurements were performed on heating from room temperature up to 425 K. After the given temperature had been reached, the sample was kept at constant temperature for about 15 minutes before the experiments started. The experiments were performed at ambient conditions. The humidity was about 80%.

## Electronic supplementary material

Supplementary Information
