# Supplementary material for: Evolution of poled state in P(VDF-TrFE)/(Pb,Ba)(Zr,Ti)O3 composites probed by temperature dependent Piezoresponse and Kelvin Probe Force Microscopy
Source: Sci Rep. 2018 Jan 10;8:378. doi: 10.1038/s41598-017-18838-1 (PMC5762672; doi:10.1038/s41598-017-18838-1)
Supplement: Supplementary file 1 — Supplementary Information [file 41598_2017_18838_MOESM1_ESM.pdf]

Supplementary Information for

Evolution of poled state in P(VDF-TrFE)/(Pb,Ba)(Zr,Ti)O3 composites probed by temperature dependent Piezoresponse and Kelvin Probe Force Microscopy

V. V. Shvartsman,[1] D. A. Kiselev,[2,4] A. V. Solnyshkin,[3,4] D. C. Lupascu,[1] and M. V. Silibin[4]

[1]Institute for Material Science and Center for Nanointegration Duisburg-Essen (CENIDE), University of Duisburg-Essen, Universitätsstraße 15, 45141 Essen, Germany

[2]National University of Science and Technology "MISiS", 119049 Moscow, Leninskiy pr. 4, Russia

[3]Department of Condensed Matter Physics, Tver State University, 170100 Tver, Russia

[4]National Research University of Electronic Technology "MIET", Bld. 1, Shokin Square, 124498 Moscow, Russia

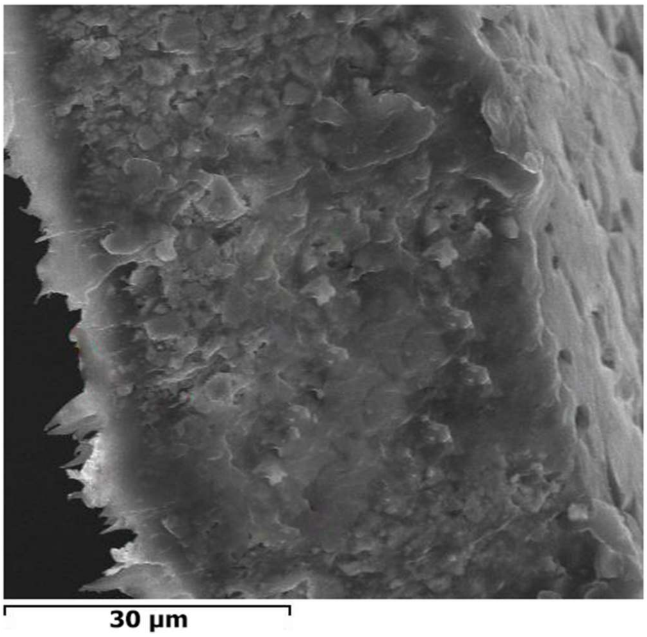

Figure S1. Scanning electron microscopy image of the P(VDF-TrFE) + 40 vol% BPZT composite film.

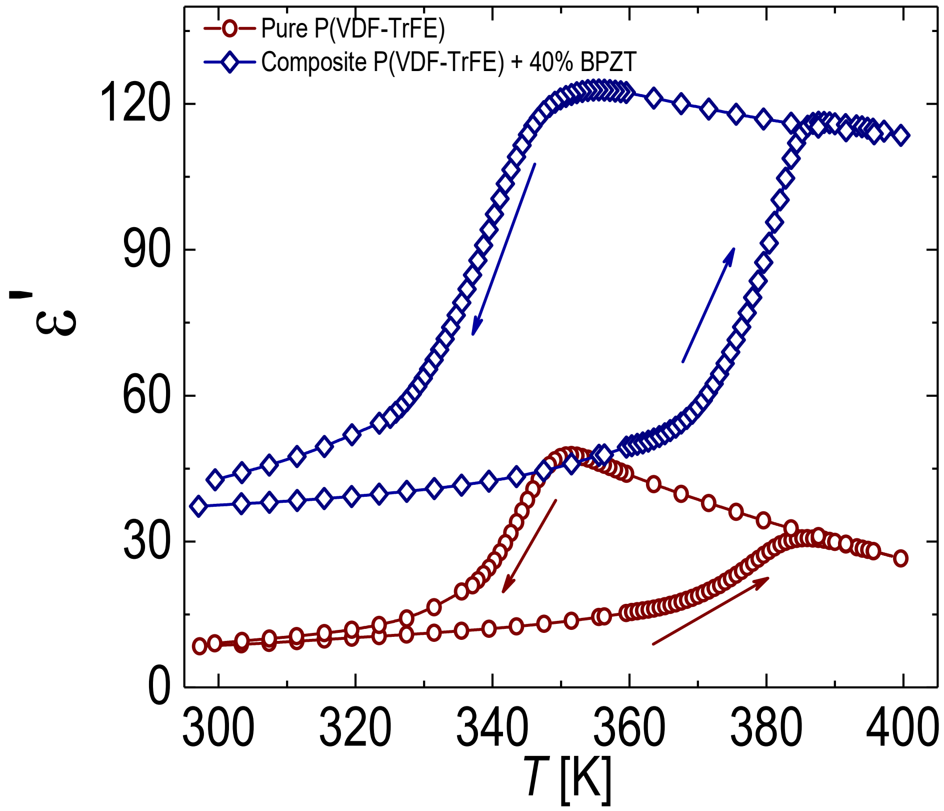

Figure S2. Temperature dependences of the dielectric permittivity of the pure P(VDF-TrFE) film and the composite film with 40 vol% of BPZT. The probing frequency is 1 kHz.
